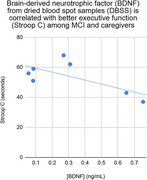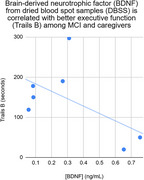# Brain‐derived neurotrophic factor and cognitive function: Piloting dried blood spot sampling as a novel method in clinical trials for mild cognitive impairment

**DOI:** 10.1002/alz70856_106286

**Published:** 2026-01-09

**Authors:** Brian Cohen, Naomi Goldsmith, Craig Story, Vadim Yerokhin, Paul Arciero, Valerie Needham, Stella Panos, Gary Warner, Cay Anderson‐Hanley

**Affiliations:** ^1^ Union College, Schenectady, NY, USA; ^2^ Gordon College, Wenham, MA, USA; ^3^ Abraxas Labs, Tulsa, OK, USA; ^4^ Skidmore College, Saratoga Springs, NY, USA; ^5^ iPACES, Clifton Park, NY, USA; ^6^ iPACES @Pacific Brain Health Center, Santa Monica, CA, USA; ^7^ UCLA, Los Angeles, CA, USA; ^8^ University of Rochester, Rochester, NY, USA; ^9^ Neuroscience Program, Union College, Schenectady, NY, USA

## Abstract

**Background:**

Clinical trials to ameliorate mild cognitive impairment (MCI) and stave off Alzheimer's and related dementias (ADRDs) can benefit from facile methods for tracking biomarker correlates linking interventions (e.g., exercise) with neuropsychological outcomes (e.g., executive function). Brain‐derived neurotrophic factor (BNDF) has been found to be related to cognitive function (Shamida et al., 2014), and to mediate the impact of exercise interventions (Leckie et al., 2014). Serum is standard for measuring BDNF, but dried blood spot sampling (DBSS) was piloted with older adults to increase sample collection in a national RCT conducted remotely during the COVID pandemic (affording self‐collection in the comfort of home with return by mail). While two pediatric studies have reported analyzable BDNF via DBSS correlating with executive function (Ghassabian et al., 2017; Skogstrand et al., 2019), this is the first known report with older adults.

**Method:**

Pilot analyses of DBSS samples were from MCI patients and caregivers (*n* = 7; ave age = 73). Participants were enrolled in a clinical trial to evaluate the neuropsychological benefit of long‐term use of a pedal‐n‐play neuro‐exergame: iPACES (an interactive Physical and Cognitive Exercise System). Executive function was assessed at baseline via videoconference: Stroop C (interference trial; 40 stimuli) and Trails B (alternating numbers and letters). Participants collected their own DBSS and returned them by mail where they were stored (‐18C). Eluants from the extraction of each 3 mm punch from DBSS cards were frozen at −80°C until analysis for BDNF (quantified using standard ELISAs).

**Result:**

Measures of executive function (Stroop C and Trails B) were found to correlate moderately with BDNF (*r* = ‐.69 and *r* = ‐.56; see Figures), such that higher levels of BDNF were associated with better cognitive performance (faster response times).

**Conclusion:**

Feasibility of at‐home DBSS collection was shown for older adult participants (including MCI) in a remote clinical trial and pilot analyses of DBSS cards returned in the mail yielded sufficient BDNF from punches for analyses. Furthermore, higher levels of BDNF correlated with better executive function thus providing preliminary validation of this novel approach. Results should be replicated in a larger sample.